# Non-hematopoietic deficiency of proprotein convertase subtilisin/kexin type 9 deficiency leads to more severe anemia in a murine model of sickle cell disease

**DOI:** 10.1038/s41598-020-73463-9

**Published:** 2020-10-05

**Authors:** J. Venugopal, J. Wang, C. Guo, H. Lu, Y. E. Chen, D. T. Eitzman

**Affiliations:** Department of Internal Medicine, Cardiovascular Research Center, University of Michigan, 7301A MSRB III, 1150 W Medical Center Drive, Ann Arbor, MI 48109 USA

**Keywords:** Diseases, Medical research

## Abstract

Proprotein convertase subtilisin/kexin type 9 (PCSK9) deficiency leads to lower cholesterol and is associated with reduced vascular complications in the general population. Cholesterol lowering may also have beneficial effects in sickle cell disease (SCD). The objective of this study was to determine effects of PCSK9 deficiency in a mouse model of SCD. Bone marrow transplantation (BMT) was performed from donor SCD mice to wild-type, PCSK9-deficient, and LDLR-deficient recipients to generate SCD controls (*Pcsk9*^+*/*+^, SCD^bmt^) with preserved PCSK9 status, SCD mice with deficiency of PCSK9 (*Pcsk9*^*−/−*^, SCD^bmt^), and SCD mice with deficiency of LDLR (*Ldlr*^*−/−*^, SCD^bmt^). Although cholesterol levels were lower in *Pcsk9*^*−/−*^, SCD^bmt^ mice compared to *Pcsk9*^+*/*+^, SCD^bmt^ mice, anemia was more severe in *Pcsk9*^*−/−*^, SCD^bmt^ mice. Increased reticulocytosis, enhanced ex vivo erythrocyte sickling, and increased erythrocyte phosphatidylserine exposure was also observed. Livers, spleens, and kidneys contained increased iron in *Pcsk9*^*−/−*^, SCD^bmt^ mice compared to *Pcsk9*^+*/*+^, SCD^bmt^ mice consistent with greater hemolysis. SCD mice with deficiency of LDLR (*Ldlr*^*−/−*^, SCD^bmt^ mice) had similar anemia as *Ldlr*^+*/*+^, SCD^bmt^ mice despite higher serum cholesterol. In conclusion, deficiency of PCSK9 is associated with worsened anemia in SCD mice due to increased hemolysis. These findings may have implications for lipid-lowering strategies in patients with SCD, as well as for potential novel modifiers of anemia severity.

## Introduction

Sickle cell disease (SCD) is one of the most common inherited blood disorders in humans with millions affected worldwide^1^. Vascular complications of SCD include pain crises, priapism, acute chest syndrome, multi-organ failure and stroke^2–4^. Although vascular complications are common, the clinical course of SCD is highly variable suggesting the importance of modifying factors^3^. Circulating lipids are important modifiers of chronic inflammatory diseases in the general population^5^, although the effect of circulating lipids on SCD phenotypes is unclear. Patients with SCD have been shown in some studies to have low cholesterol compared to a matched control group^6^. Circulating lipids may play a role in erythrocyte membrane homeostasis^7^. It is unknown whether the observed decrease in circulating cholesterol in SCD patients potentiates or ameliorates negative SCD phenotypes, or if it is merely a byproduct of hemolysis-induced increase in erythropoiesis with no affect on SCD-related anemia or organ damage.

Proprotein convertase subtilisin/kexin type 9 (PCSK9) is a protease produced primarily from the liver that targets the low density lipoprotein (LDL) receptor for degradation^8^. Although effects of genetic mutations on PCSK9 functionality is complex^9^, in general, humans with gain-of-function PCSK9 mutations have hyperlipidemia^8^ while those with loss-of-function mutations have low lipid levels^10^. Mice with genetic deficiency of PCSK9 also have reduced circulating lipid levels^11^. Circulating lipids may play a role in erythrocyte membrane homeostasis^7^ and may also modulate inflammatory pathways^12–14^.

Mouse models of SCD have been developed that mimic the predominant features of SCD in humans^15–17^. In general, these mice exhibit hemolysis, anemia, splenomegaly, and multi-organ infarcts^15–17^. SCD mice have thus been valuable towards identification of mechanisms involved in microcirculatory vaso-occlusion and in the testing of potential therapeutic interventions^18^. Generation of SCD mice with complete deficiency of a candidate gene in sufficient numbers with age and sex-matched controls through intercrosses is difficult and time consuming because of issues with multiple mutant genes and infertility in SCD mice^16,19^. We have previously generated SCD mice by bone marrow transplantation with complete penetrance of the SCD phenotype in all recipients^20,21^. Since the bone marrow is sufficient to generate SCD, and PCSK9 is produced primarily by non-hematopoietic cells, the BMT strategy is convenient to test the effects of lipid lowering in SCD. As PCSK9 is primarily produced in the liver, transplantation of SCD marrow into recipient mice should produce SCD mice with no circulating PCSK9 and hypocholesterolemia. This model was therefore implemented to determine if decreased circulating lipids was beneficial towards the anemia of SCD and related vascular phenotypes.

## Results

### PCSK9, cholesterol, and anemia in SCD mice with and without PCSK9 deficiency

**Figure 1:**
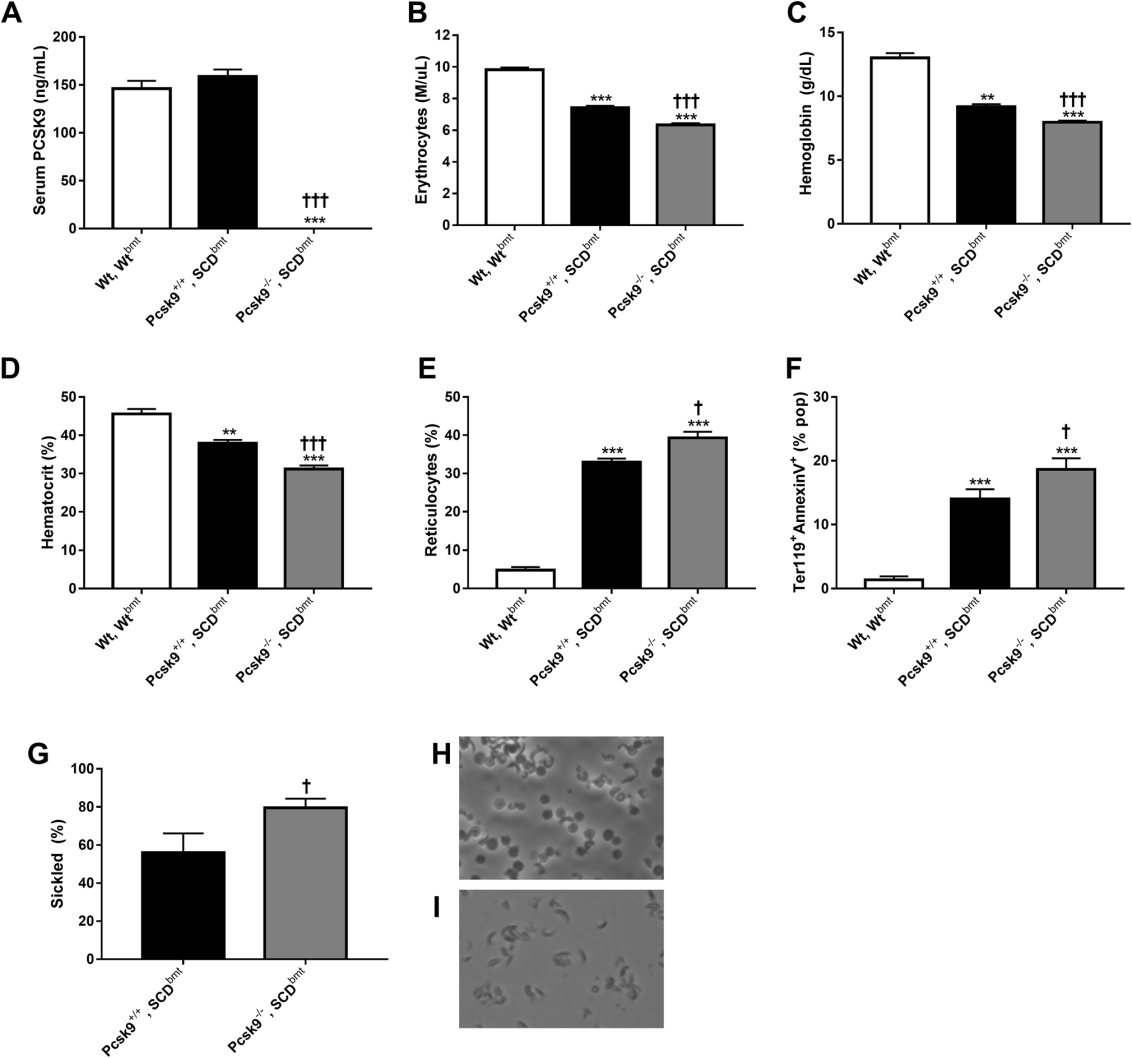
PCSK9 levels and hematologic parameters in WT mice and SCD mice, with and without *Pcsk9* deficiency. (**A**) Concentrations of circulating PCSK9 in *Wt*, Wt^bmt^, *Pcsk9*^+*/*+^, SCD^bmt^, and *Pcsk9*^*−/−*^, SCD^bmt^ mice (n = 5, each group). Circulating blood count data for (**B**) erythrocyte number (**C**) hemoglobin (**D**) hematocrit (n = 5, 14, and 12 for *Wt*, Wt^bmt^, *Pcsk9*^+*/*+^, SCD^bmt^, and *Pcsk9*^*−/−*^, SCD^bmt^ mice, respectively). (**E**) Quantification of circulating reticulocytes by new methylene blue staining (n = 9, each group). (**F**) Percent population of whole blood which stained dually with Ter119-APC-Cy7 and Annexin V-PE. (n = 9, each group) (**G**) Percentage of erythrocytes that sickled at 90 min post-introduction of 2% sodium metabisulfite; n = 4 and n = 5, for *Pcsk9*^+*/*+^, SCD^bmt^, and *Pcsk9*^*−/−*^, SCD^bmt^ mice, respectively. Representative images erythrocytes sickling from (**H**) *Pcsk9*^+*/*+^, SCD^bmt^ and (**I**) *Pcsk9*^*−/−*^, SCD^bmt^ at the 90 min time point. Error bars denote SEM. Asterisks indicate significance to *Wt*, Wt^bmt^: * < 0.05, ** < 0.01, *** < 0.005, **** < 0.001; Crosses indicate significance between *Pcsk9*^+*/*+^, SCD^bmt^ and *Pcsk9*^*−/−*^, SCD^bmt^ mice using the same scale.

To generate a model of SCD with low cholesterol levels, bone marrow was transplanted from SCD donor to PCSK9 deficient or WT recipients to generate the experimental *Pcsk9*^*−/−*^, SCD^bmt^ mice and *Pcsk9*^+*/*+^, SCD^bmt^ controls. Serum levels of PCSK9 were undetectable in *Pcsk9*^*−/−*^, SCD^bmt^ mice and within a normal range for *Pcsk9*^+*/*+^, SCD^bmt^ mice confirming that circulating PCSK9 is derived from non-hematopoietic organs (i.e. liver) (Fig. 1A). PCSK9 deficiency was associated with lower levels of total cholesterol (78.5 ± 2.23 mg/dL in *Pcsk9*^+*/*+^, SCD^bmt^ mice vs 55.74 ± 2.57 mg/dL in *Pcsk9*^*−/−*^, SCD^bmt^ mice, p < 0.01). HDL-cholesterol was lower in mice with hematopoietic PCSK9 deficiency (*Pcsk9*^+*/*+^, SCD^bmt^ vs *Pcsk9*^*−/−*^, SCD^bmt^: 19.94 ± 1.55 vs 12.33 ± 3.37, p < 0.01).

Anemia was evident in all mice receiving bone marrow from an SCD donor, however, anemia was more severe in mice lacking PCSK9 (Fig. 1B–D). Reticulocyte percentages were higher in PCSK9 deficient mice (Fig. 1E) suggesting anemia was not due to impaired erythropoiesis but rather increased hemolysis. A significant decrease in mean corpuscular volume (MCV) was observed in *Pcsk9*^*−/−*^, SCD^bmt^ erythrocytes relative to *Pcsk9*^+*/*+^, SCD^bmt^ (50.35 ± 0.57 vs 53.9 ± 1.14 fL, p < 0.005) while mean corpuscular hemoglobin and mean corpuscular hemoglobin concentration were not different (data not shown). Total circulating leukocyte counts were significantly decreased in PCSK9 deficient SCD recipients (*Pcsk9*^+*/*+^, SCD^bmt^ vs *Pcsk9*^*−/−*^, SCD^bmt^ : 47.57 ± 2.99 vs 35.38 ± 3.24, p < 0.05), with neutrophils being the only subset of leukocytes significantly altered in PCSK9 deficient SCD recipients (*Pcsk9*^+*/*+^, SCD^bmt^ vs *Pcsk9*^*−/−*^, SCD^bmt^ : 13.98 ± 1.46 vs 8.61 ± 1.03, p < 0.005).

In recipients of wild-type bone marrow, no significant differences in hematological parameters (*Pcsk9*^+*/*+^, Wt^bmt^ mice: RBC: 9.95 ± 0.16 M/uL , HB: 12.70 ± 0.21 g/dL, HCT 45.48 ± 0.60% vs *Pcsk9*^*−/−*^, Wt^bmt^ mice: RBC: 9.10 ± 0.22 M/uL, HB: 12.06 ± 0.20 g/dL, HCT: 42.70 ± 1.19%, p = NS) were observed between wild-type and PCSK9 deficient mice. However, PCSK9 deficiency in recipients of wild-type bone marrow had significantly lower levels of total cholesterol (90.58 ± 1.91 mg/dL in *Pcsk9*^+*/*+^, Wt^bmt^ mice vs 63.90 ± 3.23 mg/dL in *Pcsk9*^*−/−*^, Wt^bmt^ mice, p < 0.01), LDL-cholesterol (38.60 ± 12.31 mg/dL in *Pcsk9*^+*/*+^, Wt^bmt^ mice vs 23.37 ± 7.94 mg/dL in *Pcsk9*^*−/−*^, Wt^bmt^ mice, p < 0.01), and HDL-cholesterol (*Pcsk9*^+*/*+^, Wt^bmt^ vs *Pcsk9*^*−/−*^, Wt^bmt^ : 24.56 ± 2.28 vs 18.15 ± 2.22, p < 0.05).

### Effect of PCSK9 deficiency on erythrocyte PS exposure and sickling

PS exposure is correlated with anemia in SCD, representing premature erythrocyte senescence^22^. We analyzed PS exposure on erythrocytes by flow cytometry using an anti-Ter119-APC/Cy7 antibody and Annexin-PE. The percent population of cells which was dually stained was significantly increased in all recipients of SCD^bmt^ relative to WT^bmt^ recipients, with *Pcsk9*^*−/−*^, SCD^bmt^ mice having even greater PS exposure in Ter119^+^ cells than *Pcsk9*^+*/*+^, SCD^bmt^ mice (Fig. 1F). To determine whether the tendency of erythrocytes to sickle was increased in the absence of PCSK9, an ex vivo sickling assay was performed. At 90 min following incubation, a higher percentage of erythrocytes underwent sickling in *Pcsk9*^*−/−*^, SCD^bmt^ mice compared to *Pcsk9*^+*/*+^, SCD^bmt^ mice (Fig. 1G–I).

### Effect of PCSK9 deficiency on tissue iron deposition

**Figure 2:**
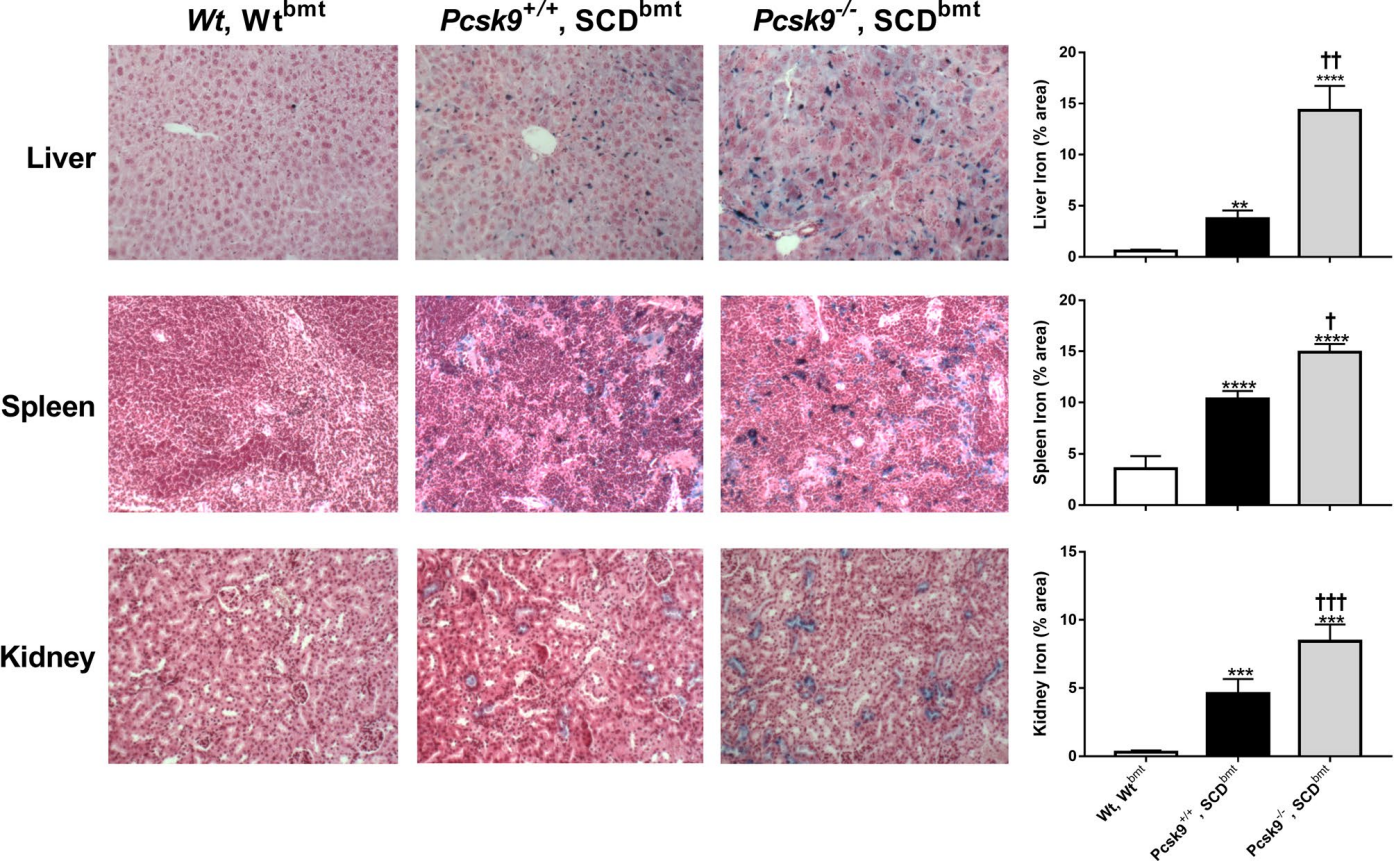
Iron deposition in tissues from WT mice and SCD mice, with and without *Pcsk9* deficiency. Representative images of iron staining indicated by blue color in liver, spleen, and kidney sections in *Wt*, Wt^bmt^, *Pcsk9*^+*/*+^, SCD^bmt^, and *Pcsk9*^*−/−*^, SCD^bmt^ mice taken at 10x. Bar graphs indicate the quantification of liver, spleen, and kidney sections between groups (n = 9). Error bars denote SEM. Asterisks indicate significance to *Wt*, Wt^bmt^: * < 0.05, ** < 0.01, *** < 0.005, **** < 0.001; Crosses indicate significance between *Pcsk9*^+*/*+^, SCD^bmt^ and *Pcsk9*^*−/−*^, SCD^bmt^ mice using the same scale.

Tissue iron deposition involving the liver, spleen and kidney was also increased in *Pcsk9*^*−/−*^, SCD^bmt^ mice compared to *Pcsk9*^+*/*+^, SCD^bmt^ mice, consistent with increased hemolysis (Fig. 2). Tissue iron deposition in the liver was also assessed in *Wt*^bmt^ mice with no observed differences in the percent area stained (0.046 ± 0.004 *Pcsk9*^+*/*+^, Wt^bmt^ vs 0.054 ± 0.008% in *Pcsk9*^*−/−*^, Wt^bmt^, p = NS).

### Tissue levels of PCSK9

Given the increased sickling we observed in *Pcsk9*^*−/−*^, SCD^bmt^ mice, we considered the possibility that PCSK9 may be regulating a receptor on the erythrocyte. If true, we would expect to detect PCSK9 associated with erythrocytes in *Pcsk9*^+*/*+^, SCD^bmt^ mice. To test this possibility, erythrocytes were isolated from 500 uL of whole blood, washed, lysed, and then PCSK9 was measured by ELISA from the erythrocyte lysate. However, PCSK9 was undetectable in erythrocyte lysates, suggesting PCSK9 was not directly associated with erythrocytes. Since PCSK9 is synthesized and also internalized with the LDL receptor by the hepatocyte^23–25^ , we concurrently assessed the expression of PCSK9 in livers from *Wt*, Wt^bmt^, *Pcsk9*^+*/*+^, SCD^bmt^, and *Pcsk9*^*−/−*^, SCD^bmt^ mice. Hepatocyte PCSK9 protein was observed in *Wt*, Wt^bmt^ and *Pcsk9*^+*/*+^, SCD^bmt^ mice (9.50 ± 1.53 and 10.83 ± 2.09 pg/mg lysate, respectively) but was undetectable in *Pcsk9*^*−/−*^, SCD^bmt^ mice, as expected.

### Effect of LDLR deficiency on cholesterol levels and anemia in SCD mice

The predominant mechanism by which PCSK9 affects cholesterol levels is through targeting the hepatic LDL receptor for degradation^23–25^. To determine whether the deficiency of LDLR would improve anemia in SCD mice, *Ldlr*^*−/−*^, SCD^bmt^ mice were generated by bone marrow transplantation of sickle cell marrow to LDLR deficient recipients. Total cholesterol levels were higher in *Ldlr*^*−/−*^, SCD^bmt^ compared to *Ldlr*^+*/*+^, SCD^bmt^ mice (153.91 ± 4.84 vs 80.84 ± 2.73 mg/dL, p < 0.001). LDL-cholesterol was significantly increased in *Ldlr*^*−/−*^, SCD^bmt^ mice relative to *Ldlr*^+*/*+^, SCD^bmt^ mice (143.00 ± 6.48 vs 19.77 ± 3.27 mg/dL, p < 0.005) while HDL-cholesterol was unaltered (20.56 ± 1.29 vs 19.94 ± 1.55 mg/dL, p = NS). However, anemia (erythrocytes: 6.24 ± 0.13 vs 6.56 ± 0.25 M/uL, p = NS) and reticulocytosis (31.72 ± 1.97 vs 31.78 ± 0.73%, p = NS) were not affected. Despite no difference in anemia, hepatic iron staining was decreased in *Ldlr*^*−/−*^, SCD^bmt^ mice relative to *Ldlr*^+*/*+^, SCD^bmt^ mice (1.00 ± 0.10 vs 3.73 ± 0.69, p < 0.05).

## Discussion

Sickle cell disease shows a high level of phenotypic heterogeneity. For example, in the Cooperative Study of SCD in the USA^3^, 39% of 3578 patients had no episodes of pain but 1% had more than 6 pain episodes per year. These findings suggest the presence of modifying genetic and environmental factors that remain to be elucidated. Mouse models of sickle cell disease have been developed that share many features of SCD with humans and have been successfully used to identify genetic and pharmacologic interventions that modify the SCD phenotype. Interventions targeting cellular adhesive interactions have shown to be beneficial in both preclinical and clinical studies of SCD^20,26^. In the non-SCD population, lipid lowering therapies reduce these adhesive interactions^27^ and also protect against vascular complications such as myocardial infarction and stroke^28^. Although the mechanism(s) for vascular events in SCD and non-SCD patients are likely to be distinct, lipid lowering therapy could provide beneficial effects in SCD. However, patients with SCD have been shown in some studies to have lower cholesterol levels than non-SCD patients^6^ and lipids may play a role in erythrocyte membrane homeostasis^7^. Thus, there may be both risks and benefits to lipid lowering in SCD. Intriguingly, two short-term pilot trials investigating the effect of statin treatment in SCD patients have reported positive responses to treatment^29,30^. While statins are potent cholesterol lowering agents in humans, their effect on lipids in mice are controversial with limited or no effect on cholesterol in some studies^27^ Therefore, a genetic intervention with a gene deficiency state associated with low cholesterol in both humans and mice is attractive to test the effect of cholesterol lowering in SCD mice. Although lipid metabolism is different between mice and humans^31^, PCSK9 has been shown to regulate cholesterol levels through effects on LDL receptors in both mice and humans^32^.

In this study, a successful model of SCD with lowered levels of cholesterol was generated by transplanting bone marrow from SCD mice to recipient mice deficient in PCSK9. Levels of PCSK9 were undetectable in the plasma and livers of *Pcsk9*^*−/−*^, SCD^bmt^ mice and this was associated with reduced levels of cholesterol. While we hypothesized that erythrocyte turnover might be reduced through beneficial vascular effects of lipid lowering, we found more severe anemia in mice lacking PCSK9. This could be the result of impaired erythropoiesis due to a lipid requirement by the bone marrow for cell production. However, our finding of increased reticulocytosis with worsened anemia in *Pcsk9*^*−/−*^, SCD^bmt^ mice compared to *Pcsk9*^+*/*+^, SCD^bmt^ mice suggests that increased hemolysis was contributing to more severe anemia. The increased serum cholesterol in *Ldlr*^*−/−*^, SCD^bmt^ mice relative to *Ldlr*^+*/*+^, SCD^bmt^ mice did not improve hematopoietic parameters, which suggests these PCSK9-mediated affects are mediated through a LDLR-independent target protein.

Since erythrocyte lipid homeostasis could be altered by cholesterol lowering^7^, especially in a chronic hemolytic state like SCD where erythrocyte production is greatly increased, we determined whether the PCSK9 deficiency state conferred enhanced erythrocyte sickling. In an ex vivo sickling assay, we found that sickling of erythrocytes was increased in *Pcsk9*^*−/−*^, SCD^bmt^ mice compared to *Pcsk9*^+*/*+^, SCD^bmt^ mice, consistent with this hypothesis. We also observed a decrease in erythrocyte MCV in *Pcsk9*^*−/−*^, SCD^bmt^ mice compared to *Pcsk9*^+*/*+^, SCD^bmt^ mice without any alteration in MCH. Decreased MCV in SCD has been postulated to increase sickling rates due to increased likelihood of interactions between hemoglobin molecules^33^. Additionally, erythrocyte phosphatidylserine exposure, as determine by Annexin V staining, was increased in SCD compared to WT mice and even further increased in *Pcsk9*^*−/−*^, SCD^bmt^ mice compared to *Pcsk9*^+*/*+^, SCD^bmt^ mice. Phosphatidylserine exposure is correlated to anemia in SCD, representing premature erythrocyte senescence^22^. Although fetal hemoglobin levels are very low in adult SCD mice, we cannot rule out an effect of PCSK9 on lowering fetal hemoglobin levels further.

Tissue iron deposition has been shown to correlate with the severity of hemolytic anemia and tissue hemolysis in SCD^34^. We have previously shown that a treatment promoting erythrocyte stability with reduced hemolysis leads to reduced iron deposition in kidneys of SCD mice^21^. The worsened anemia due to PCSK9 deficiency was associated with increased iron staining in liver, spleen, and kidney sections. Although other mechanisms regulating iron deposition in SCD could be affected by PCSK9, this finding is consistent with increased erythrocyte turnover. Although *Ldlr*^*−/−*^, SCD^bmt^ mice did not have altered anemia or reticulocytosis compared to controls, iron deposition in their livers was decreased relative to control livers, indicating that pathways involved in lipid metabolism may affect hepatic iron accumulation in hemolytic states.

PCSK9 is largely produced by the liver and, consistent with this, PCSK9 was not detectable in plasma or in hepatic lysates from *Pcsk9*^*−/−*^, SCD^bmt^ mice, even though bone marrow was derived from a *Pcsk9*^+*/*+^ donor. The precise mechanisms by which PCSK9 deficiency affects SCD erythrocytes is unknown in this study. PCSK9 could lead to reduced hepatic production of a circulating lipid that is important for erythrocyte stability in SCD, however, no PCSK9 was detected in erythrocyte lysates suggesting the erythrocyte is an indirect target or that PCSK9 regulates a surface erythrocyte protein without internalization or tight association. It is also possible that PCSK9 could affect other cell types, including platelets^35^, which could affect the SCD phenotype^36^.

In conclusion, we have shown that PCSK9 deficiency leads to worsening of the SCD phenotype in mice. Additional studies are necessary to uncover the underlying mechanisms responsible for this phenotype and the effects on vascular complications. PCSK9 may be a genetic modifier of SCD phenotype and caution should be used if PCSK9 inhibitors are considered for lipid lowering therapy in patients with SCD.

## Methods

### Animals

Male C57BL6/J (wild-type, WT), homozygous SCD (*SCD,* *Hbb*^*hβs/hβs*^) along with corresponding wild-type SCD control (*Wt*, *Hbb*^+*/*+^), homozygous PCSK9 deficient (*Pcsk9*^*−/−*^) and homozygous LDLR deficient (*Ldlr*^*−/−*^) mice were purchased from Jackson Laboratory (Bar Harbor, Maine, USA), stock numbers #000664, #013071, #002014, #005993 and #002207, respectively. The homozygous PCSK9 deficient (*Pcsk9*^*−/−*^) and homozygous LDLR deficient (*Ldlr*^*−/−*^) mice were originally generated through the manipulation of 129-derived embryonic stem cells, which were then injected into C57BL6/J blastocysts. Each strain was then backcrossed into the C57BL6/J strain for greater than 10 generations. SCD mice with and without recipient PCSK9 or LDLR deficiency were generated by bone marrow transplantation (BMT) from Hbb^hβs/hβs^ or wild-type (Hbb^+/+^) donors to WT (*Wt*, SCD^bmt^ or *Wt*, Wt^bmt^), PCSK9 deficient (*Pcsk9*^*−/−*^, SCD^bmt^ or *Pcsk9*^*−/−*^, Wt^bmt^), or LDLR deficient (*Ldlr*^*−/−*^, SCD^bmt^) recipients. Mice were housed under specific pathogen-free conditions in static microisolator cages with tap water ad libitum in a temperature-controlled room with a 12:12-h light/dark cycle. Mice were fed a standard laboratory rodent diet (No. 5001, TestDiet, Richmond, IN, USA). All animal use protocols complied with the Principle of Laboratory and Animal Care established by the National Society for Medical Research and were approved by the University of Michigan Committee on Use and Care of Animals.

### Bone marrow transplantation

SCD mice were generated by BMT as previously described^20,21^. Briefly, 8 week-old male WT, *Pcsk9*^*−/−*^ and *LDLR*^*−/−*^ mice were used as recipients that received bone marrow from SCD or WT male donors. Bone marrow was harvested from the donor mice by flushing their femurs and tibias with RPMI medium (Gibco/Invitrogen, Carlsbad, CA) containing 10% fetal bovine serum (Gibco/Invitrogen, Carlsbad, CA). Cells were then centrifuged at 300 g and resuspended in phosphate-buffered saline before injection. Each recipient mouse was irradiated (2 × 650 rad [0.02 × 6.5 Gy]) and then injected with 4 × 10^6^ bone marrow cells via the tail vein in a 200 μL bone marrow suspension in phosphate-buffered saline. Acid water (6 mM HCl, pH = 2.5) was provided to animals beginning 4 days before BMT to 4 weeks following BMT. Fifteen weeks following BMT, complete blood counts were performed with a Hemavet (Drew Scientific, Inc) on whole blood collected in EDTA-lined tubes via retro-orbital sampling from isofluorane-anesthetized mice. Reticulocyte percentages were quantified by new methylene blue staining (Ricca Chemical Company, Arlington, TX), according to manufacturer's instructions and expressed as a percentage of total erythrocytes.

### Equipment and settings

Images for tissue iron analysis and the sickling assay were acquired on a Nikon Microphot-SA (Nikon Instruments Inc, Melville, NY; #15941) set on brightfield illumination at ambient room temperature, using a MicroPublisher3.3RTV camera (Teledyne Qimaging, Surrey, BC Canada; #Q25984) and MetaMorph software (Molecular Devices, LLC, San Jose, CA). All illumination settings were set and then maintained at the same illumination for the duration of the imaging of all slides. Images were converted to JPG using ImageJ, (National Institutes of Health, Bethesda, MD).

### Tissue iron staining

Similar to the manner previously described^21^, mice were sacrificed at 20 weeks post-BMT, the bodies perfused with PBS, then the livers, spleens and kidneys were fixed in neutral-buffered formalin, and then dehydrated in 90% and then 70% ethanol, embedded in paraffin, and 5-μm thick sections cut. Iron deposition was determined with the Iron Stain Kit (Sigma-Aldrich, St Louis, MO; Cat# HT20) according to manufacturer’s instructions. Three random fields of each section were photographed at 10 × and then the percent area stained was calculated with automated computer software (ImageJ, National Institutes of Health, Bethesda, MD).

### Sickling assay

The ex vivo sickling assay was performed as described previously^37^. Under isofluorane anesthesia, blood was drawn retro-orbitally into EDTA-lined tubes and 5 uL of whole blood was placed into 5 uL of 2% sodium metabisulfite on a glass slide. A coverslip was added and 2 random fields of view were taken at 20 × for each slide at 90 min. The number of sickled erythrocytes and total erythrocytes were each quantified per view to determine the percent of erythrocytes sickled.

### Phosphatidylserine (PS) exposure on erythrocytes

At the time of sacrifice, 30 uL of the whole blood collected via cardiac puncture was fixed in 100 uL of a solution containing 1% formalin and 0.01% glutaraldehyde in PBS, and stored at 4 °C until use. To assess PS exposure, 10 uL of the fixed cell suspension was aliquoted into new centrifuge tubes, washed with PBS, and blocked in 3% BSA for 1 h. Fixed cells were then resuspended in a 3% BSA PBS solution containing a 1:500 dilution of both anti-Ter119 conjugated to APC/Cy7 antibody (Biolegend; Cat# 116223) and Annexin-PE (BD Pharmingen; Cat# 556421), and incubated for 30 min at room temperature protected from light. Cells were washed with PBS and analyzed using a Gallios flow cytometer (Beckman Coulter, Indianapolis, IN, Model #B5-R1-V2). 50,000 events were recorded for each sample. Dual labeling of Ter119 and Annexin was detected and expressed as percentage of total population of cells. Quadrant gates were set based on unlabeled and singly labeled samples, and the gates were applied universally to each sample. Log scale was used for both Ter119 and Annexin V. No compensation was used. Data was analyzed with Kaluza Acquisition Software (Beckman, Coupter, Indianapolis, IN).

### Measurement of PCSK9 and cholesterol

A murine PCSK9 enzyme-linked immunosorbent assay was performed according to manufacturer’s instructions (R&D Systems, Inc.; Minneapolis MN, USA). Blood for ELISA was collected via cardiac puncture at the time of sacrifice and plasma prepared by centrifugation at 8500 rpm for 10 min. For cell-associated PCSK9, the pelleted erythrocytes were washed, then lysed with distilled water. Livers were dissected, flash frozen, and then homogenized using a Tissue Tearor (Biospec Products Inc, Bartlesville, OK). Protein concentration of erythrocyte and liver lysates were determined via BCA kit (Thermo Fisher Scientific, Waltham, MA, USA). ELISA values were normalized to protein concentration for each sample. Total cholesterol was measured using the Infinity Cholesterol kit using Data-Trol Normal Control as a known control value (both from Thermo Fisher Scientific, Waltham, MA, USA). Commercial kits were used to assess LDL and HDL cholesterol and compared to the HDL/LDL Cholesterol Calibrator Set (all from Diazyme Laboratories, Poway, CA, USA).

### Statistical analysis

Values are expressed as mean ± standard error of the mean. The alpha level for each test was 0.05. A one-way analysis of variance (ANOVA) was used to determine statistical significance, followed by Dunnett’s multiple comparisons test. The Brown-Forsythe test and Bartlett’s test was used to determine whether group variances were equal and normally distributed. If data was determined to have equal group variance but not be normally distributed, a Kruskal Wallis H test was performed, followed by Dunnett’s multiple comparisons test. The statistical significance of differences between two groups was determined by the Student’s 2-tailed t test.

## Data Availability

All data generated during this study are included in this manuscript.
